# Weeding out variability: a proof-of-concept for producing uniform F_1_ hybrid *Cannabis sativa* L. using single-seed descent

**DOI:** 10.1093/hr/uhag038

**Published:** 2026-02-19

**Authors:** Lennard Garcia-de Heer, Jos Mieog, Adam Burn, Matthew Nolan, Lei Liu, Stephen Manansala-Siazon, Tobias Kretzschmar

**Affiliations:** Faculty of Science and Engineering, Southern Cross University, Lismore, NSW 2480, Australia; Faculty of Science and Engineering, Southern Cross University, Lismore, NSW 2480, Australia; Faculty of Science and Engineering, Southern Cross University, Lismore, NSW 2480, Australia; Faculty of Science and Engineering, Southern Cross University, Lismore, NSW 2480, Australia; Faculty of Science and Engineering, Southern Cross University, Lismore, NSW 2480, Australia; Faculty of Science and Engineering, Southern Cross University, Lismore, NSW 2480, Australia; Faculty of Science and Engineering, Southern Cross University, Lismore, NSW 2480, Australia

## Abstract

*Cannabis sativa* is a wind-pollinated, predominantly dioecious, and outcrossing crop associated with high levels of genetic variability even within a single cultivar. As such, seed-grown crops are often constrained by variability issues, decreasing production efficiency and product consistency. F_1_ hybrid seed technology offers great potential to address these limitations by generating genetically uniform populations from a cross of two inbred parental lines. In *C. sativa,* single-seed descent (SSD) is currently the most viable method to produce these homozygous parental lines necessary for F_1_ hybrid seed production. This study exemplifies the potential of SSD coupled with chemically induced sex reversion to produce fully homozygous lines and its subsequent application in creating five F_1_ hybrid accessions. Up to six rounds of SSD were performed in an 18-month period on 16 different lines, highlighting the speed of methodology. Inbreeding through XY males was most successful and offered the greatest advantages of the lines assessed. The F_1_ hybrid lines were statistically more uniform than the inbred or original lines and more vigorous than the inbred lines, with F_1_ lines increasing seed yield between 3.9% and 155% when compared to their midparents indicating the potential to exploit heterosis. Chemotype stability was achieved in some F_1_ hybrid lines, showing that seed-grown cannabinoid crops would be possible in some contexts using F_1_ hybrid methodology, paving the way for the validation of this breeding technique in field settings and highlighting a path toward commercial hybrid seed systems in *C. sativa.*

## Introduction

*Cannabis sativa* is primarily dioecious, with a long history of cultivation for food, fiber, and pharmaceuticals [1]. *Cannabis sativa* is a diploid species with an XX/XY sex chromosome system with XX individuals generally expressing female reproductive traits and XY individuals expressing male reproductive traits [2]. Due to the large size of the Y chromosome, genome size differs between XX and XY individuals, 1636 and 1683 Mbp respectively [3]. Sex expression is strongly influenced by photoperiod, with XY males generally growing more vigorously early and completing development sooner [4]. *Cannabis sativa* has a flexible sexual system, and sporadic hermaphroditic flowers are common. XX monoecious varieties have been developed to improve seed and fiber uniformity [5], while an XY monoecious accession was recently identified [6]. In pharmaceutical contexts, females are valued for cannabinoid-rich glandular trichomes on flowers and bracts, whereas males provide higher-quality fiber [7, 8]. The cannabinoid profile is largely determined by the expression of synthases encoded by multigene families with variable copy number and high allelic diversity [9]. The major cannabinoids are cannabidiolic acid (CBDA), tetrahydrocannabinolic acid (THCA), and their precursor cannabigerolic acid (CBGA). Five chemotypes are recognized: Type I (THCA dominant), Type II (1:1 THCA/CBDA), Type III (CBDA dominant), Type IV (CBGA dominant), and Type V (lacking cannabinoids) [10–13].

As a wind-pollinated, predominant out-crosser, seed-grown *C. sativa* is highly variable. Currently, most *C. sativa* hybrid seeds originate from pairwise crosses between individuals with high heterozygosity, yielding inconsistent offspring and limited seed quantities, more suited for breeding rather than large-scale production [14]. This inconsistency has driven widespread adoption of clonal propagation in the medicinal cannabis industry, where chemical uniformity is essential. Yet, maintaining stock plants is resource intensive and increases vulnerability to pests and diseases [15], and the performance of successive generations of clonal crops reduces over time [16]. In contrast, true F_1_ hybrid seed is created from crossing two unrelated homozygous lines creating genetically and phenotypically uniform offspring with high heterosis [17]. Although some breeders have released F_1_ hybrids for medicinal markets, production remains limited and adoption incomplete. To our knowledge, only a few attempts have been made to develop true F_1_ hybrid technology for broad acre cropping of *C. sativa* by private companies*,* and the underlying methodologies remain unclear [18]*.*

In broad-acre cultivation, variability in developmental timelines remains a major challenge for *C. sativa*. Inconsistent floral timing can reduce pollination efficiency [19], while optimal harvest windows for mixed-sex fiber crops are unclear [7]. Many crops, such as wheat and rice, achieve phenotypic uniformity through self-fertilization-induced inbreeding, which increases homozygosity and stabilizes traits [17]. This is more complex in naturally outcrossing species like *C. sativa* [20], which is also often more susceptible to inbreeding depression [21]. Uniform, vigorous plants grown from F₁ hybrid seed are therefore a key goal in production. F₁ hybrids, produced by crossing genetically distinct homozygous parents, yield high-performing, genetically consistent offspring and have been central to plant breeding for over a century, notably in rice and maize [22–24]. Advances such as cytoplasmic male sterility and declining production costs are increasing F₁ hybrid adoption among growers [25]. Generating homozygous parental lines is critical to this process, and such lines are valuable not only for breeding but also for genomic research and development applications including mutagenesis, quantitative trait loci analysis, gene isolation, cloning, and genome mapping [26].

Inbreeding fixes desirable traits by increasing homozygosity across generations. In *C. sativa*, serial inbreeding via single-seed descent (SSD) is currently the most practical route to produce true homozygous parental lines, which can then be crossed to generate uniform F₁ seed [27]. Techniques to induce doubled haploids, effective in other crops, have thus far been unsuccessful in *C. sativa* [28]. SSD, however, is slow and laborious, typically requiring six generations to approach functional homozygosity [29], and may be further constrained by infertility and declining agronomic performance arising from inbreeding depression.

Dioecious species would generally rely on sib-mating or bi-parental inbreeding to increase homozygosity, though fixation occurs more slowly than with self-fertilization, raising time and cost [30]. However, the *C. sativa’s* capacity for sex reversion enables SSD inbreeding through effective selfing in dioecious lines [31]. Sex reversion, the induction of flowers of the opposite sex, can occur at any stage of the life cycle via environmental or chemical triggers. Most commonly, genetically female plants are induced to produce male flowers using the ethylene inhibitor silver thiosulfate (STS) [32], while the converse, female flowers on males, can be induced using the ethylene promoter ethephon (ETH) [33]. Both the induced male flowers and induced female flowers are fertile and are key to produce sex-specific seed [34, 35]. Additionally, naturally occurring monoecious *C. sativa* lines are capable for self-fertilization without the need for chemical treatments.

Inbreeding often causes phenotypic abnormalities and low survival in naturally outcrossing plants by reducing fitness through two genetic mechanisms. First, it increases homozygosity, raising the likelihood that (partially) recessive deleterious alleles are expressed [21]. In outcrossing populations, these alleles are typically masked in heterozygotes, but with greater homozygosity, they phenotypically manifest, reducing fitness. Second, inbreeding reduces heterozygosity at loci where heterozygotes have a selective advantage, a phenomenon known as overdominance or heterozygote advantage [21]. At such loci, loss of heterozygosity directly decreases fitness. Together, these processes underpin inbreeding depression, though their relative impact varies among species and populations. Thus, while SSD and sex reversion provide pathways to generate homozygous *C. sativa* lines, programs must balance trait fixation against reduced vigor and abnormal phenotypes.

Consistency of sex expression is critical in this breeding approach, both as an economically valuable trait and to maintain fertility across generations. Concerns have been raised that inbreeding *C. sativa* alters sex expression and reduces fertility through inbreeding depression, potentially limiting the feasibility of achieving homozygosity via serial self-fertilization [36]. Anecdotal reports from the cannabis community suggest inbreeding increases hermaphroditic flowers, reduces vigor, and causes fertility loss. Empirical support comes from two studies that observed reduced biomass after a single round of self-pollination, although neither pursued full homozygosity nor assessed fertility outcomes [37, 38]. The only study exploring the effects of multiple rounds of self-fertilization did not provide genetic estimates of changes in heterozygosity in the inbred lines (IBLs); therefore, it remains unclear whether the resulting hybrid lines are true F1 hybrids [18]. Moreover, published studies to date have focused on STS-induced self-fertilization of female plants, with no corresponding exploration of XY inbreeding [18, 37, 38]. Consequently, evaluation of inbreeding’s impact on sex expression and fertility in *C. sativa* remains lacking, representing an important avenue for future research.

This study aimed to generate homozygous lines through SSD inbreeding to determine whether sex expression and fertility are altered across successive generations, and whether outcomes differ among dioecious XX, dioecious XY, and monoecious XX and XY individuals. As a proof of concept for true F₁ hybrid seed production, five F₁ hybrid lines derived from four successfully developed homozygous IBLs were assessed by comparing the vigor and uniformity of the F₁ hybrids with their inbred parental lines and the original heterozygous accessions.

## Results and discussion

### Inbreeding increases the propensity for floral abnormalities

Inbreeding via SSD and self-fertilization was trialed across 16 accessions, including 6 XX monoecious, 1 XY monoecious [6], 1 XY dioecious, 2 XX dioecious, and 3 lines inbred on both the XX and XY sides (Table 1). Between one round and six rounds of self-fertilization were completed between April 2021 and October 2023 aided by STS- and ETH-induced sex reversion. Further inbreeding of some lines was halted due to poor seed set (Juso_14 and Syrian) or very slow flowering and seed development (Fibrimon and Forose). Five lines were successfully advanced to the S_6_ generation [Bernberger (Bern), Eltham Dio Australis (EDA), Futura, IPK_36, and No. 8_XX], while progress with the remaining lines (French, Kongo Hanf, No. 8_XY, Romanian, and SI-1) was constrained by time and resources, highlighting the practical limitations of SSD in *C. sativa* across diverse germplasm.

**Table 1:** Summary of inbreeding attempts.

Accession	Origin	Collection	Flowering type	Sex chromosomes	Successful attempts	Failed attempts	Mean flowering length (days)
Bern	Germany	IPK	Monoecious	XX	6	0	51
EDA	Australia	Kavasil	Monoecious	XX	6	1	51
Fibrimon	Germany	IPK	Monoecious	XX	1	0	68
Forose	Unknown	IPK	Monoecious	XX	1	0	68
IPK_37 (French)	France	IPK	Dioecious	XX	2	0	74.5
Futura	Germany	IPK	Monoecious	XX	6	0	54.5
IPK_CAN_36 (IPK_36)	Unknown	IPK	Monoecious	XY	6	0	61.5
Juso 14	Germany	IPK	Monoecious	XX	3	3	63
Kongo Hanf	Spain	IPK	Dioecious	XX	2	0	78.5
No. 8	Australia	Kavasil	Dioecious	XX	6	4	73
No. 8	Australia	Kavasil	Dioecious	XY	4	0	54
IPK_32 (Romanian)	Romania	IPK	Dioecious	XX	3	3	63
IPK_32 (Romanian)	Romania	IPK	Dioecious	XY	4	1	42
SI-1	China	Kavasil	Dioecious	XY	2	0	85.5
IPK_57 (Syria)	Syria	IPK	Dioecious	XX	1	1	87
IPK_57 (Syria)	Syria	IPK	Dioecious	XY	0	1	NA

Germplasm originates from the IPK collection and the proprietary collection of Kavasil Pty Ltd. In each attempt, after sex detection, the most vigorous seedling was isolated in a pollen-proof chamber and treated with 4.3 mM ETH and/or 6 mM STS solutions.

**Figure 1:**
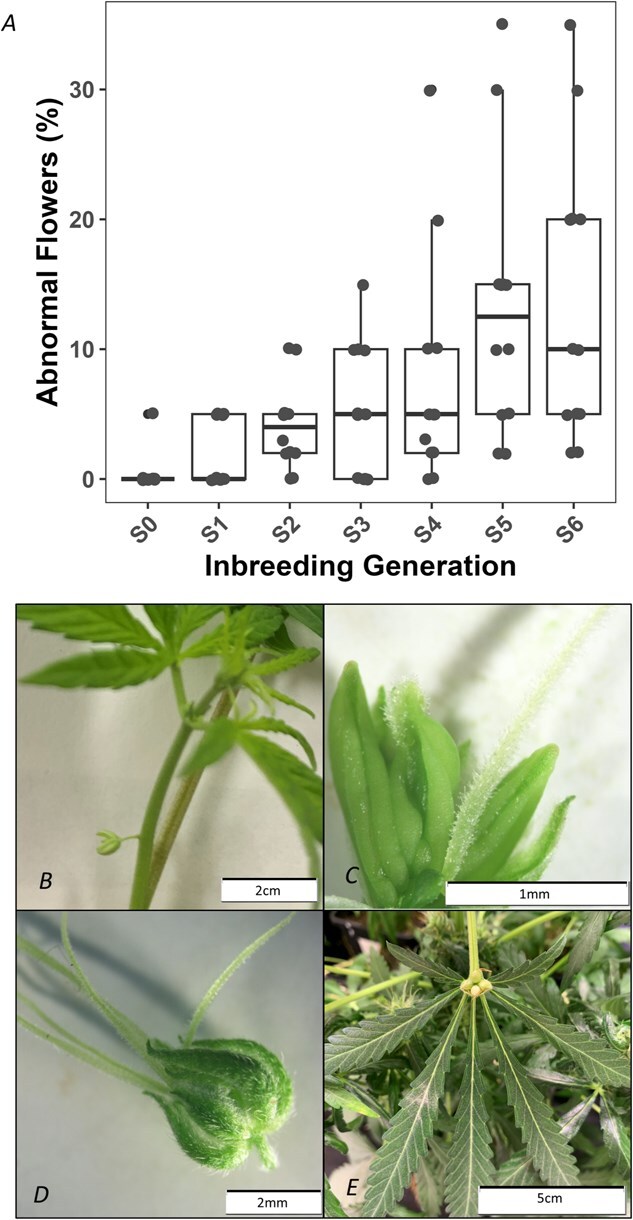
Effect of inbreeding on the propensity to produce abnormal flowers in IPK_36, (A) proportion of abnormal flowers in each generation at anthesis, refer to Table S2 for *post hoc* groupings, (B) male flower growing from an internode, (C) intermediate sex organ, anther with a style growing out the tip, (D) hermaphroditic flower, male perianth with multiple stigmas, (E) single flower containing four immature seeds growing from the leaf base.

In *C. sativa*, typical male flowers hang from a thin pedicel with five tepals and five stamens [39], while typical female flowers are sessile and enclosed by a perigonal bract covering a single ovary with a style terminating in two outward-protruding stigmas [40]. Floral abnormalities (any deviation from this) and a concurrent reduction in seed yield were observed in all lines from the fourth generation of self-fertilization onward. Monoecious accessions appeared to produce abnormal flowers more frequently than dioecious lines, although all lines exhibited some degree of aberration. To quantify any potential increases in floral abnormalities observed while performing the inbreeding, all seven generations (*n* = 12) of a single line (IPK_36) were grown side by side. Figure 1A illustrates the propensity for floral abnormalities at anthesis when all seven generations of IPK_36 were grown together in a common-garden experiment, with the proportion of abnormal flowers increasing from 0.4% in S₀ to 13.7% in S_6_. Traits directly linked to reproductive success, such as floral morphology, are known to be particularly sensitive to inbreeding depression [41]. The observed phenotypic abnormalities included spatial (flowers arising from internodes or leaf bases) and morphological deviations. Dissection revealed altered sex organ development, with ovaries ranging from 0 to 5, anthers 0 to 7, styles 0 to 5, and the presence of intermediate anther/style organs (Fig. 1B–E). These findings align with reports of inbreeding depression affecting pistil and stamen shape and position in *Prunus dulcis* and *Michelia coriacea* [42, 43], and reduced flower size in *Solanum carolinense* [44], with floral abnormalities often decreasing fertility, and subsequent seed set [45]. Such effects are more pronounced in predominantly outcrossing species like *C. sativa*, indicating that IBLs are unlikely to be suitable for commercial production, as is traditionally the case with predominantly autogamous crops such as *Solanum lycopersicum* [46].

Issues with floral abnormalities and reduced fertility from S_4_ onward were mitigated by treating different sections of the plants with both the ethylene promoter ETH and the ethylene inhibitor STS. This facilitated the production of fertile seed, indicating that inbreeding depression was impacting hormone signaling. Overdominance has been reported to positively influence endogenous gibberellin and brassinosteroid levels [47, 48], while heterosis has been shown to decrease salicylic acid and ethylene levels in *Arabidopsis* [49, 50]. Because ethylene strongly affects sex expression in *C. sativa* [35, 51] and seed set was rescued by the combined application of STS and ETH, the increased occurrence of abnormal flowers with progressive inbreeding may be driven by inbreeding-induced alterations in ethylene signaling.

### Creating IBLs is easier for monoecious lines and XY males offer advantages over XX

Pollen abundance and seed set were limiting factors throughout inbreeding, particularly in the later generations of dioecious female lines, where induced male flowers often produced very little pollen, causing more XX inbreeding attempts to fail (8 of 22 attempts; Table 1). While this may reflect the specific accessions used in this study, inbreeding is consistently reported to reduce pollen performance, often resulting in smaller pollen grains and slower pollen tube growth [52]. Additionally, the effectiveness of female-to-male sex reversion in *C. sativa* is genotype dependent [15]. Consequently, inbreeding through dioecious XX females may require stabilizing a line’s capacity for sex reversion if present as a segregating trait, potentially increasing the risk of unwanted spontaneous male flowers. These issues were largely avoided in XX monoecious lines, which showed higher success rates (3 of 6 to S_6_) compared with dioecious females (1 of 5 to S_6_; Table 1). XX monoecious lines were easier to manage, as chemical treatments were not always required and pollen fertility appeared less affected by inbreeding (4 of 27 attempts failed). Unlike XY male lines, sex selection at the seedling stage was unnecessary after the first round of inbreeding. However, XX monoecious lines exhibited the slower developmental timeline typical of XX plants.

In contrast, ovule fertility was largely maintained in ETH-treated males, and pollen abundance and viability were less affected. Abnormal female flowers frequently still set seed (Fig. 1E), resulting in a lower failure rate in the XY lines evaluated in this study (2 of 12 attempts; Table 1). This aligns with the well-established phenomenon of sex-specific inbreeding depression, where the homogametic sex often experiences stronger effects [41]. However, in dioecious *C. sativa*, where sex reversion is required, the impact of inbreeding is likely influenced by additional factors beyond the gametic composition of an individual.

Developmental timelines of males and females remained consistently different despite ETH and STS treatments, with XY plants reaching seed maturity more rapidly. Consistent with the previously reported monoecious male phenotype [6], generating IBLs through XY males offers a distinct advantage by producing both XX and XY offspring, enhancing end-use flexibility. All inbreeding attempts with the IPK_36 monoecious male line were successful, suggesting that this trait could be advantageous if introgressed into broader genetic backgrounds to facilitate more efficient inbreeding.

Thus, each sexual phenotype presents distinct advantages and limitations (Table 2). We suggest that generating IBLs in dioecious *C. sativa* via XY individuals is preferable where feasible, as this approach combines a lower risk of failure, shorter developmental timelines, and greater end-use flexibility. However, the failure rates may be an artifact of the experimental conditions or genotypes used in this study, and a comprehensive comparison of XX and XY self-fertilization is warranted. Nevertheless, inbreeding monoecious phenotypes remains technically easier than dioecious lines.

**Table 2:** Summary of the advantages and disadvantages of inbreeding through each sexual phenotype.

Sexual phenotype	Advantages	Disadvantages
Dioecious female	Only one round of sex selection. Feminized seed is desired in cannabinoid/seed production. Easier targeting of female traits.	Pollen viability issues, fixing a plant’s ability to produce hermaphroditic flowers. Always requires chemical treatments. Slower developmental timeline.
Monoecious female	Only one round of sex selection. Chemical treatments not always required.	Slower developmental timeline. Limited end use of IBL
Dioecious male	Fast developmental timeline. Better pollen viability. Flexible end use. Easier targeting of male traits.	Sex selection required every round. Always requires chemical treatments.
Monoecious male	Fast developmental timeline. Better pollen viability. Flexible end use. Easier targeting of male traits. Chemical treatments not always required.	Sex selection required every round.

### Evaluating sex expression of inbred progeny

#### Progeny sex expression after inbreeding XX monoecious lines

**Figure 2:**
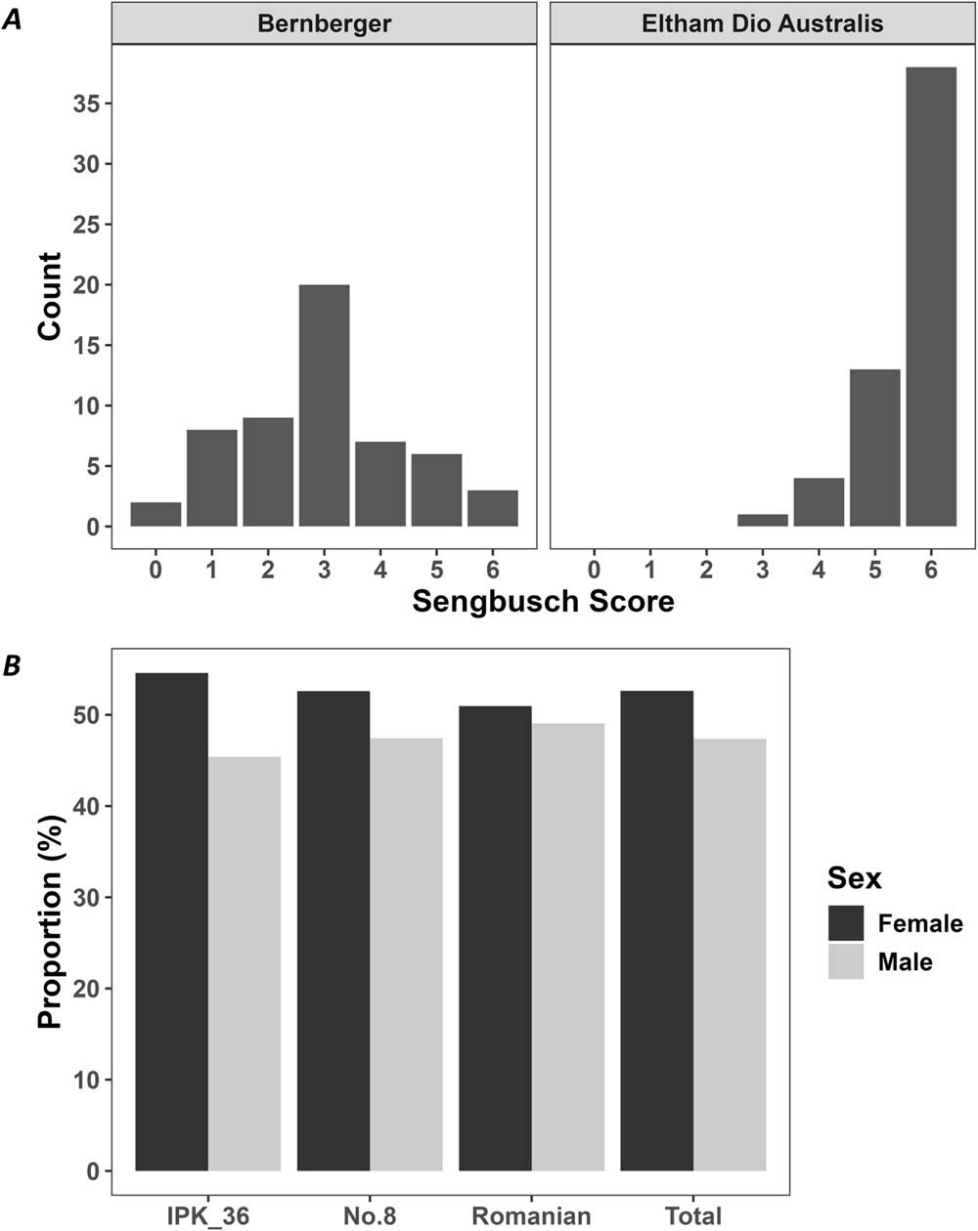
XX monoecious and XY male inbred offspring sex expression, (A) histogram of modified Sengbusch score of two S_1_ XX monoecious accessions Bern (*n* = 55) and EDA (*n* = 56) after 5 weeks of flowering, 0 = 100% male flowers, 1 = 99%–81% male flowers, 2 = 80%–61% male flowers, 3 = 60%–41% male flowers, 4 = 40%–21% male flowers, 5 = 20%–1% male flowers, 6 = 100% female flowers [54], all offspring were determined to have an XX chromosomal makeup by PCR (Fig. S2), (B) bar chart of the proportion (%) of male and female offspring of three self-pollinated XY lines. IPK_36 (*n* = 185), No. 8 (*n* = 135), and Romanian (*n* = 210), this equates to a 1:1 M/F offspring ratio by χ^2^ (*P* = .22).

The sex expression of progeny after a single round of self-fertilization (S_1_) in XX monoecious lines Bernberger (Bern) and EDA was assessed (Fig. 2A). After 5 weeks of flowering, seedlings from both lines produced male and female flowers in a compound raceme, as expected for XX monoecious plants. The proportion of male-to-female flowers were scored in the progeny using the modified Sengbusch scale [53] (Fig. 2A). Polymerase chain reaction (PCR) analysis of a subset of monoecious plants, including the two with 100% male flowers, confirmed that all carried XX sex chromosomes (Fig. S2). These results support previous findings that inflorescence structure is more closely associated with sex chromosome composition than with the sex of individual flowers [35].

The relative proportion of male and female flowers in monoecious *C. sativa* is a plastic trait, readily influenced by environmental conditions [55]. However, the distinctly different distributions on the Sengbusch scale observed for the two XX monoecious varieties in this study, grown under uniform conditions, suggest the influence of a major gene on this trait (Fig. 2A). Similarly, in monoecious cucurbits, the proportion of male and female flowers is primarily controlled by tissue-specific expression of a single rate-limiting ethylene biosynthesis gene [56,57]. The causal variants underlying the genetic control of monoecy in *C. sativa* have not yet been identified, and further investigation is required, although potential sex determining genes are being identified [58].

These results suggest that, although still variable after one round of inbreeding (Fig. 2A), the relative proportion of male and female flowers in *C. sativa* could potentially be stabilized through successive inbreeding. This conclusion is further supported by the assessment of F₁ hybrid lines in this study (see flowering traits section below) and an approach already used to produce F₁ hybrid spinach varieties [59]. Moreover, the observed differences in flower ratios between lines indicate a more complex inheritance pattern than simple Mendelian dominance, consistent with previous hypotheses regarding monoecy in *C. sativa* [31].

#### Progeny sex expression after inbreeding XY lines

We assessed the sex of progeny from the first round of male (XY) inbreeding in the Romanian, No. 8, and IPK_36 lines (Fig. 2B). Assuming Mendelian inheritance from self-pollination of an XY individual, and that YY plants exhibit a male phenotype as observed in other species [60,61], progeny are expected to segregate at a 3:1 male-to-female ratio if YY plants are viable, or 2:1 if YY plants are completely lethal [62]. We specifically investigated the potential existence of YY individuals in *C. sativa*, as Charlesworth [63] suggested that YY plants are more likely in species where the Y chromosome has not undergone degeneration, as is the case in *C. sativa*, and viable YY plant crossed with an XX female would enable the production of masculinized XY seed.

Chi-square tests comparing observed phenotypes to expected ratios (3:1, 2:1, and 1:1 male-to-female ratios) indicated that the data were consistent with a 1:1 ratio (*P* = .22). Using larger sample sizes and multiple genotypes, this corroborates Toth’s [64] previous findings of a 1:1 male-to-female ratio in self-fertilized XY *C. sativa*. Toth proposed that this pattern may result from ovules carrying a Y chromosome being nonviable, which could also explain the low seed germination rates observed in this study (Table S4). Overall, the sex expression of inbred XY progeny provides no evidence for the existence of viable YY plants in *C. sativa*.

### Inbreeding causes variable decrease in heterozygosity

**Figure 3:**
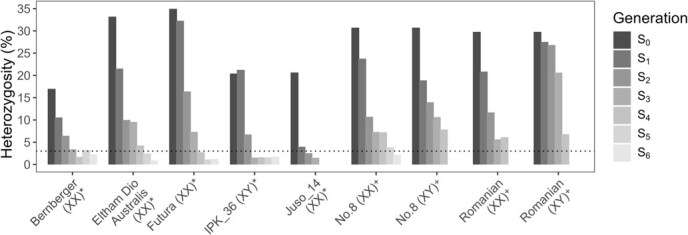
Summary of heterozygosity in each generation of IBLs with three or more generations, leaf samples were taken the day plants were introduced to flowering photoperiod, heterozygosity was estimated using a 1500 SNP panel and functionally homozygous was considered reached in individuals with <3% heterozygosity, chromosomal makeup of each IBL in brackets, * indicates monoecious flowering, and ^+^ indicates dioecious.

Heterozygosity was estimated in all lines with three or more successful round of inbreeding by calculating the proportion of heterozygous alleles using a 1500-SNP HASCH panel, illustrating the effectiveness of SSD in generating homozygous IBLs (Fig. 3). Initial heterozygosity rates ranged from 0.17 in Bern to 0.35 in Futura (Fig. 3). The magnitude of change in heterozygosity between successive generations was highly variable, highlighting the importance of employing robust methods for heterozygosity estimation when performing SSD.

### F_1_ hybrid lines are more uniform than original lines and IBLs with heterosis evident

The adoption of F₁ hybrid varieties has steadily increased since their introduction over a century ago [25]. Although commercial seed companies have recently begun offering F₁ hybrid *C. sativa* varieties for the cannabinoid market in limited quantities, to our knowledge, this study represents the first published account of the underlying methodology.

**Figure 4:**
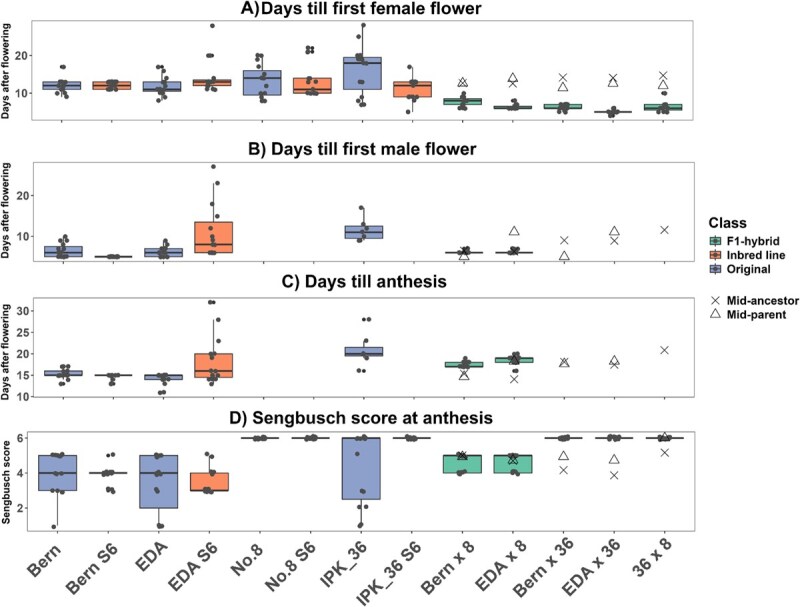
Comparison of flowering traits of original (blue), IBL (orange), and F_1_ hybrid (green) lines (*n* = 15), (A–C) the number of days was counted from when the photoperiod was reduced to 12:12 from 18:6, (C) anthesis was scored when the first male flower opened and released pollen, (D) qualitative 0–6 scale adapted from Neuer and Sengbusch (1943) [54], with 0 = 100% male flowers and 6 = 100% female flowers, variance between lines was assessed via Levene’s test and differences in means by Kruskal–Wallis test, refer to Table S3 for statistical groupings and Table 3 for the parentage of F_1_ hybrid lines.

**Figure 5:**
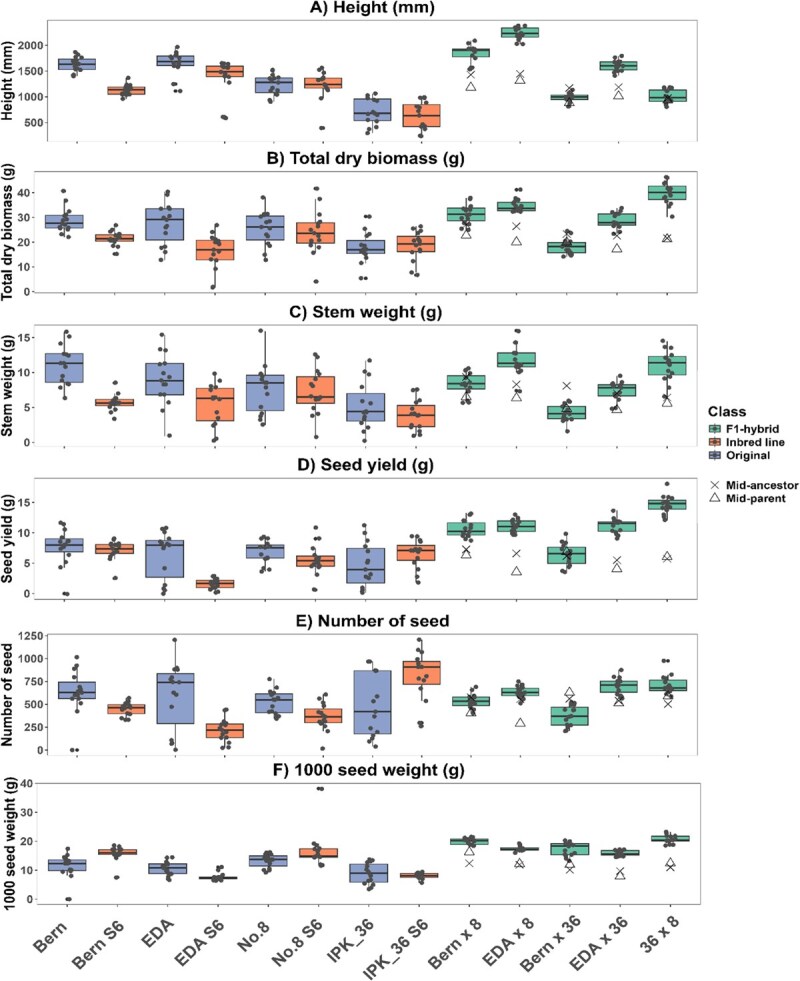
Comparison of harvest traits of original (blue), IBL (orange), and F_1_ hybrid (green) lines (*n* = 15), (A) height was measured at harvest before the whole plant was dried, (B-C) after reaching uniform dryness, stems were removed before flower and leaf material were passed through a series of sieves to collect seed, (D-F) seed was then counted and used to calculate 1000 seed weight, variance between lines was assessed via Levene’s test and differences in means by Kruskal–Wallis test refer to Table S3 for statistical groupings and Table 3 for the parentage of F_1_ hybrid lines.

**Figure 6:**
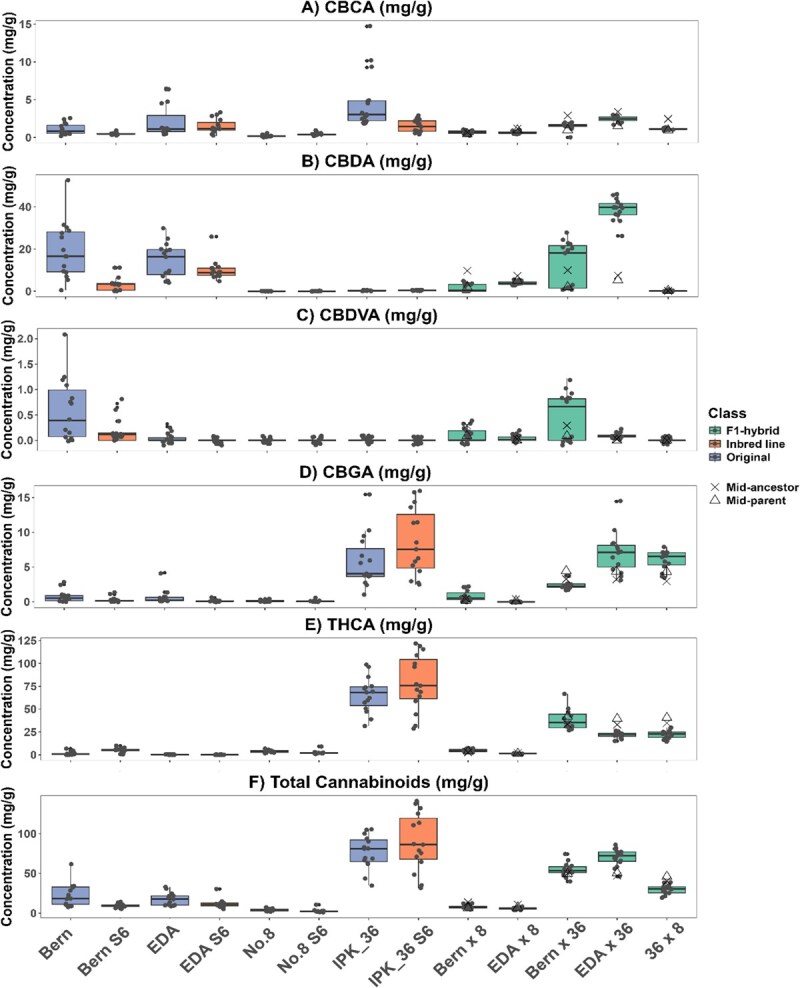
Comparison of major cannabinoid concentrations (mg/g) of original (blue), IBL (orange), and F_1_ hybrid (green) lines (*n* = 15), (A) (CBCA), (B) CBDA, (C) CBDVA, (D) CBGA, (E) THCA, (F) Total cannabinoids, variance between lines was assessed via Levene’s test and differences in means by Kruskal–Wallis test, refer to Table S3 for statistical groupings and Table 3 for the parentage of F_1_ hybrid lines.

Differences between lines were evident across all measured traits when comparing original, IBL, and F₁ hybrid lines (Figs 4–6), likely reflecting underlying genotypic variation and heterogeneity, with both variance and means differing significantly (Levene’s test, *P* < 0.01; Table S3). For clarity, measured variables were grouped into sex expression and flowering traits (Fig. 4), harvest traits (Fig. 5), and chemical traits (Fig. 6).

#### Sex expression and flowering

Onset of flowering and sexual maturity was accelerated in all F₁ hybrid lines compared to both original and IBL groups (Fig. 4A and B), with immature flowers appearing on all F₁ plants within 4 days of flowering initiation. In contrast, inbreeding had little effect on flowering timing, with original and IBLs producing flowers over 21 and 23 days after switching to short-day conditions, respectively (Table S3). Male flowering and anthesis were generally more uniform than female flowering, except in the EDA S_6_ line (Fig. 4A–C). The rapid onset of flowering of F₁ hybrid lines supports early-onset flowering as a dominant trait in *C. sativa*, consistent with other species where flowering time is often controlled by few genes; e.g. early flowering in *Daucus carota* is governed by a single dominant gene [65], while a single gene represses flowering under long-day conditions in tomato [66]. Faster maturity relative to midparent or midancestor values indicates that heterosis contributed to the increased speed of flowering after switch to short-day conditions in these lines (Fig. 4).

Notably, differences in sex expression, measured using the modified Sengbusch score at anthesis, were observed between lines (Fig. 4D). Inbreeding significantly reduced variability in the proportion of male and female flowers (*P* < 0.001) in monoecious IBLs, and this pattern persisted in the F₁ hybrid lines. These results indicate that, while environmental factors influence male-to-female flower ratios in monoecious *C. sativa* [55], a strong genetic component exists that can be fixed through inbreeding, producing a narrower phenotypic range. Combined with the observation of two inbred XX monoecious lines exhibiting distinct Sengbusch distributions (Fig. 2A), this suggests potential applications in hemp seed production, where male-to-female flower ratios could be adjusted to optimize pollination and maximize seed set, as previously demonstrated in monoecious *Cucumis sativus* [67].

Female monoecy in *C. sativa* is thought to be a dominant X-linked trait [31], potentially a result of the recombination of the X and Y chromosomes [53]. This suggests that crosses between inbred XX monoecious and XX dioecious plants should produce 100% XX monoecious F₁ offspring. In this study, two (EDA × 8 and Bern × 8) were 100% XX monoecious as expected, while the other two (EDA × 36 and Bern × 36) were 100% dioecious female (Fig. 4D). The discrepancy corresponds to the seed parent genotype: No. 8 is a pure dioecious line, whereas IPK_36 produces XY monoecious plants [6]. This coincided with XY plants from the IPK_36 S_6_ IBL producing only female flowers (Fig. S3), whereas the original IPK_36 line exhibited more variable Sengbusch scores (Fig. 4D). Schilling *et al.* [55] reported that monoecious varieties grown under artificial 12:12 photoperiods, as in this study, produced a higher proportion of dioecious female plants than under natural lighting. Similarly, Gao *et al.* [67] found that pollination of female flowers increased subsequent female flower formation and suppressed male flower formation in monoecious cucumbers. Together, these findings suggest that, although the flowering conditions favored female expression, the XY monoecious trait may inhibit male flower formation in XX females. If so, this trait could be used to reduce spontaneous hermaphroditism in medicinal crops or to produce feminized F_1_ hybrid seed. Importantly, this study generated XX individuals with no female organs (Fig. 2A—Bern) and XY individuals with no male organs (Fig. S3) without chemical intervention, illustrating the extreme plasticity of sex expression in *C. sativa*.

#### Harvest traits

Vigor and biomass accumulation varied substantially among the lines tested (Fig. 5). EDA S_6_ and Bern S_6_ exhibited significantly lower total dry weight than their original counterparts, whereas No. 8 S_6_ and IPK_36 S_6_ did not differ from their respective original lines (Fig. 5). Thus, IBLs were not consistently less vigorous than the original accessions. Inbreeding generally impacts vigor more strongly in outcrossing than in self-fertilizing species due to greater evolutionary pressure in the latter to purge deleterious alleles. However, as the most vigorous seedlings were selected for self-pollination during IBL generation, these results may partly reflect the selection pressures imposed by SSD rather than the intrinsic effects of inbreeding. Nevertheless, IBLs with unchanged vigor have been produced in some outcrossing species, such as *Zea mays* [68], and this study demonstrates the potential for similar outcomes in *C. sativa,* which is consistent with previous work in this species [18].

F₁ hybrid lines were not consistently more vigorous than the original lines (Fig. 5). Only EDA × 8 and 36 × 8 exhibited larger total biomass than both original ancestor lines (Fig. 5B). This pattern was mirrored in stem dry weight (DW), used as a proxy for fiber and biomass yield, with F₁ hybrid lines not consistently producing higher stem yields.

However, all F₁ hybrid lines were larger than their direct inbred parents, except Bern × 36, indicating heterosis and suggesting the original open-pollinated lines already carried substantial heterozygosity (Fig. 3), which is additionally supported by the reduced performance of the IBL lines when compared to the original lines (Figs 4–6). Additionally, F₁ hybrids that did not show increased vigor, such as Bern × 36, may reflect noncomplementary parental combinations or the presence of heterotic groups, despite phylogenetic evidence of distant relationships between IBLs (Fig. S1) [37,69].

Seed yield was more consistently enhanced in the F_1_ hybrid lines, with four out of five lines (all but Bern × 36) yielding significantly more than their direct IBL parents or original lines (Fig. 5D, Table S3). Yield increase was primarily due to seed weight rather than seed number, as 1000-seed weight mirrored these differences (Fig. 5E and F), whereas seed number per plant did not (Fig. 5E). The improvement likely reflects the more synchronous flower development in the F_1_ hybrids (Fig. 4A–C), which promotes uniform pollination and seed maturation. Greater consistency in seed development and increased seed size may provide a practical advantage in *C. sativa* cultivation, facilitating predictable harvest timing, easier hulling, and reduced seed breakage.

F₁ hybrid lines need to consistently outperform open-pollinated varieties to be economically viable, which was not uniformly observed in this study. Several factors may explain this. First, the high heterozygosity and genetic diversity of the original lines likely limited the difference in heterosis between F₁ hybrids and originals. Second, the lack of biomass increase in some F₁ hybrids may reflect the combining ability of the IBLs, as parent compatibility is critical for exploiting heterosis [70]. Crosses between excessively distant parental lines can reduce vigor through outbreeding depression [71]. Third, the controlled environment conditions, including pot size and light height, may have restricted the full expression of heterosis. Consequently, performance traits should be evaluated in larger populations and field settings to better capture the potential of F₁ hybrid lines. However, given that seed yield was consistently increased in the F₁ hybrid lines when compared to their midancestor values (F₁ hybrid seed yield increased comparatively by 3.9% increase to 155%, Fig. 5D), this breeding methodology may be particularly suitable for industrial hempseed crops.

#### Cannabinoid stability

The therapeutic potential of cannabis is due to its ability to produce cannabinoids [72]. When grown for pharmaceutical purposes, cannabinoid uniformity is a critical consideration in the production of medicinal cannabis for therapeutic products. In Australia, the Therapeutic Goods Administration (TGA) requires that dried flower products remain within ±20% of each stated constituent [73], which has led most producers to rely on vegetative propagation [15]. Although inbreeding increased uniformity (Levene’s test, *P* > 0.001), it did not significantly alter total cannabinoid concentrations in any of the IBLs compared to their original lines (Fig. 6). Additionally, the F_1_ hybrids mostly exhibited cannabinoid concentrations intermediate between their pollen donors and seed parents as shown by their midparent values (Fig. 6, Table 3), reflecting the expected blending of parental chemical profiles [13].

**Table 3:** Summary of crosses between S_6_ IBLs to produce F_1_ hybrid varieties.

	**Pollen donor**			**Seed parent**		
**Hybrid line**	**Accession**	**Flowering type**	**Chromosomes**	**Accession**	**Flowering type**	**Chromosomes**
EDA × 8	EDA S_6_	Monoecious	XX	No. 8 S_6_	Dioecious	XX
Bern × 8	Bern S_6_	Monoecious	XX	No. 8 S_6_	Dioecious	XX
36 × 8	IPK_36 S_6_	Monoecious	XY	No. 8 S_6_	Dioecious	XX
EDA × 36	EDA S_6_	Monoecious	XX	IPK_36 S_6_	Dioecious	XX
Bern × 36	Bern S_6_	Monoecious	XX	IPK_36 S_6_	Dioecious	XX

Pairwise crosses were performed by isolating one individual from each line together in a pollen-proof chamber, the IPK_36 S_6_ pollen donor was treated with STS to increase the proportion of viable male flowers, whereas treatment was not required in all other plants for crosses to be successful.

However, only two of the five F_1_ hybrid lines (EDA × 8 and Bern × 8; Fig. 6) had all individuals within the TGA-specified range of ±20% for all quantified cannabinoids. This suggests that, although producing uniform cannabinoid seed crops within regulatory limits is possible, additional measures beyond SSD, such as marker-assisted selection, are required to stabilize the cannabinoid biosynthetic pathway. This is evident in the Bern S_6_ and Bern × 36 lines, which contain two distinct CBDA and cannabidivarinic acid (CBDVA) subpopulations (Fig. 6B). Varin dominant chemotypes have been shown to be polygenic [74], indicating that some heterozygosity remains in one of the related genes. Moreover, F_1_ hybrids that failed to achieve chemotypic stability (Bern × 36, EDA × 36, and 36 × 8; Fig. 6) were derived from crosses between parents of different chemotypes (Type I × Type III and Type I × Type V), highlighting the importance of parental selection when breeding for uniform chemotypes. Collectively, these results demonstrate that F_1_ hybrid breeding can confer cannabinoid stability [18], but achieving consistent chemotypes requires careful parental matching and, ideally, marker-assisted selection during SSD to produce IBLs suitable for targeted cannabinoid production.

## Conclusion

In this study, we demonstrated the potential to create fully inbred *C. sativa* lines (IBLs) through SSD and their application in producing F_1_ hybrid *C. sativa*. To our knowledge, this is the first report exemplifying this methodology in this species, thereby allowing the generation of valuable genetic resources for further research. The impact of inbreeding depression was variable across the lines tested, indicating that homozygosity can be achieved without severe detrimental effects in at least some cultivars, if selection for vigor is performed in each generation. The dual application of STS and ETH proved instrumental in advancing lines through successive inbred generations. Inbreeding dioecious cultivars via the male side was the most effective approach due to shorter timelines, higher success rates, and greater end-use flexibility, followed by monoecious cultivars, whereas inbreeding dioecious cultivars via the female side was the most challenging. SSD also successfully stabilized several traits, providing insights into their inheritance, particularly for monoecy, which appears to be governed by complex genetic architecture [53]. The phenotypic extremes of monoecy observed (XX females with 100% male flowers, and XY males with 100% female flowers) as well as the small range in Sengbusch scores in IBLs and F_1_ hybrid lines exemplify the potential to fix the ratio of male to female flowers depending on end use requirements.

The resulting F₁ hybrid lines showed potential for consistent increases in uniformity across all measured variables, which holds notable value for cannabinoid production. Promising effects of heterosis were observed for seed yield but results also indicated that achieving consistent results would require careful parental selections and matching. The methodology successfully exemplifies the potential of F₁ hybrid varieties and provides a foundation for future variety development and performance assessment under field conditions.

## Materials and methods

### Selection of accessions for inbreeding

Cannabis cultivation, sampling, storage, and processing were performed in strict adherence to Sections 23(4)(b) and 41(b) of the New South Wales Drug Misuse and Trafficking Act 1985, held under the Authority granted to Prof. Bronwyn Barkla of Southern Cross University (SCU), issued by the New South Wales Ministry of Health, Australia. All cannabis germplasm was obtained either from a private collection (Kavasil PTY LTD) under a specific Material Transfer Agreement or from the Leibniz Institute of Plant Genetics and Crop Plant Research [Leibniz-Institut für Pflanzengenetik und Kulturpflanzenforschung (IPK), Germany], imported under a federal Office of Drug Control (ODC) license to import No. 1820928 and handled under the Food and Agriculture Organization of the United Nations governed Standard Material Transfer Agreement (sMTA).

A total of 16 accessions were selected (Table 1) to capture a broad range of geographic origins and flowering types (Fig. S1), with particular emphasis on accessions with early flowering and fast maturity. Three dioecious lines were subjected to inbreeding through both XY and XX pathways to allow direct comparison of their developmental and reproductive differences.

### Creating IBLs

Serial SSD was performed on 16 accessions to generate the IBLs. After germination of every generation, sex was determined visually or via PCR using MADC2 primers [75]. The most vigorous individual of the desired sex was isolated in pollen-proof chambers, and flowering was induced by shifting the photoperiod from 18:6 to 12:12. At this stage, sex reversion was induced in dioecious lines to enable self-fertilization using foliar applications of STS and/or ETH on a single branch [32, 33]. Treatments were applied every 2 days, comprising three applications of 6 mM STS and/or two applications of 4.3 mM ETH. Monoecious lines were left untreated with plant growth regulators whenever possible, but they were treated when floral abnormalities became apparent.

#### PCR conditions

Reactions were carried out with 5–50 ng of genomic DNA as a template in 25 μl using Platinum Taq (Invitrogen) according to manufacturer’s protocols and 2 μM forward and reverse primers (Sigma-Aldrich). The following conditions were used: 1 cycle at 95°C for 2 min, 30 cycles of 95°C for 30 s, 52°C for 30 s, 72°C for 30 s, and 1 cycle at 72°C for 5 min in a Veriti 96-Well Thermal Cycler (Applied Biosystems). Agarose (1.2%; Benchmark Scientific) supplemented with 5-μl/100-ml GelRed (Biotium) in 0.5× tromethamine/boric acid/ethylenediaminetetraacetic acid buffer (Sigma) was used for agarose gel electrophoresis (Bio-Rad equipment) and run at 100 V for 60 min. GelDoc (Bio-Rad) was used to capture the gel image with a 1-kb DNA ladder (New England Biolabs) to estimate PCR product size.

#### Estimating heterozygosity levels

Leaf samples from all lines were collected where three or more rounds of inbreeding were successful, and DNA was extracted using the DNeasy Plant Mini Kit (Qiagen) following the manufacturer’s instructions. DNA samples were sent to a commercial genotyping service for SNP panel analysis (Diversity Arrays Technology). Heterozygosity levels were assessed using a 1500-SNP marker panel, and the mean proportion of homozygous alleles was calculated with Tassel 5 (Table S1) [76].

The same SNP panel was used to estimate accession relatedness within the germplasm collection using Tassel v5.2.94 and Darwin v6.0.21. Phylogenetic trees were constructed in MEGA11: Molecular Evolutionary Genetic Analysis version 11 [77]. Multisequence alignments were performed using the MUSCLE algorithm, and neighbor-joining trees were generated with 500 bootstrap replicates. Trees were then exported for visualization using ggtree v3.62 (Fig. S1) [78].

#### Evaluating sex expression of inbred progeny

Sex ratios of S₁ plants derived from XY and monoecious lines were determined by visual assessment. PCR was employed to evaluate the chromosomal composition of inbred monoecious plants. XX dioecious lines were not subjected to PCR, as the literature indicates that their progeny is expected to be genetically female [34]. For XY lines, assuming YY plants are fully viable, and segregation follows Mendelian principles, offspring are expected to exhibit a 3:1 male-to-female ratio, or a 2:1 ratio if YY individuals are nonviable [62]. Female monoecy is thought to be controlled by a single dominant locus on the X chromosome (X^m^X) [31].

#### Assessing impact of inbreeding depression on floral morphology

All seven inbred generations (S_0_–S_6_) of accession IPK_CAN_36 (IPK_36) produced by SSD were grown side by side to assess the effects of inbreeding depression on abnormal flower development as a proxy for reproductive capacity. Fifty seeds from each generation were germinated in 70:30 coco-coir/perlite media supplemented with Osmocote Pro-3-4M (4.3 g/l), and the 12 most vigorous seedlings were selected 7 days after germination. Plants were grown under a 12:12 light cycle (*n* = 12, 800 μmol m^−2^ s^−1^) and scored weekly for abnormal flowers and sex expression using a modified Sengbusch scale [54, 79]. Abnormal flowers were defined as any floral structures that deviated from a typical male (hanging from a thin pedicel with five tepals and five stamens) or typical female (sessile, enclosed by a perigonal bract covering a single ovary with a style terminating in two outward-protruding stigmas) arising from a node [39, 40].

### Assessing vigor and uniformity of F_1_ hybrid lines

Once IBLs were confirmed as functionally homozygous (heterozygosity <3% achieved in six IBLs, Fig. 3), pairwise crosses were performed in pollen-proof chambers using a single plant from each line to generate a series of F₁ hybrid lines (Table 3). IBLs were paired to prevent self-pollination in the seed parent.

Four original accessions (Table 1), their corresponding IBLs (Fig. 3), and five resulting F₁ hybrids (Table 3) were grown side by side to evaluate the effects of heterosis and inbreeding on vigor and uniformity. Seeds were germinated and grown vegetatively under an 18:6 photoperiod (300–500 μmol m^−2^ s^−1^) for 3 weeks, then potted into 2-l containers with hydroponic media (70% coco coir, 30% perlite) supplemented with Osmocote Pro-3-4M (4.3 g/l) and induced to flower under a 12:12 photoperiod (800–1000 μmol m^−2^ s^−1^). Plants were blocked by line to reduce shading effects at 16.7 plants m^−2^. All dioecious males were removed (from No. 8 and 36 × 8 lines), leaving monoecious (XX and XY) or dioecious females (*n* = 15 per line). Flowering traits were scored daily until ~50% male flowers were open. Flower sex was recorded when the flower primordia were identifiable as male or female. Anthesis was recorded when pollen release was visible. Excess male clones were flowered separately to ensure optimal pollination. Agronomic traits were recorded at harvest when seeds began to shatter, followed by drying in the dark (15°C, 10% RH). Once dry, seeds were threshed, biomass recorded, and flower material from the topmost inflorescence sampled for secondary metabolite analysis. Midparent and midancestor values were calculated for each trait in F₁ hybrids by averaging the values of the direct IBL parents (midparent) or the original open-pollinated lines (midancestor) to assess potential heterosis.

### Secondary metabolite analysis

Cannabinoids were quantified using the high performance liquid chromatography method described in Dimopoulos *et al.* [80].

## Supplementary Material

Web_Material_uhag038

## Data Availability

The data that support the findings of this study are available from the corresponding author (T.K.) upon reasonable request.
