# Supplementary figures and images for: Weeding out variability: a proof-of-concept for producing uniform F1 hybrid Cannabis sativa L. using single-seed descent
Source: Hortic Res. 2026 Feb 19;13(5):uhag038. doi: 10.1093/hr/uhag038 (PMC13188225; doi:10.1093/hr/uhag038)

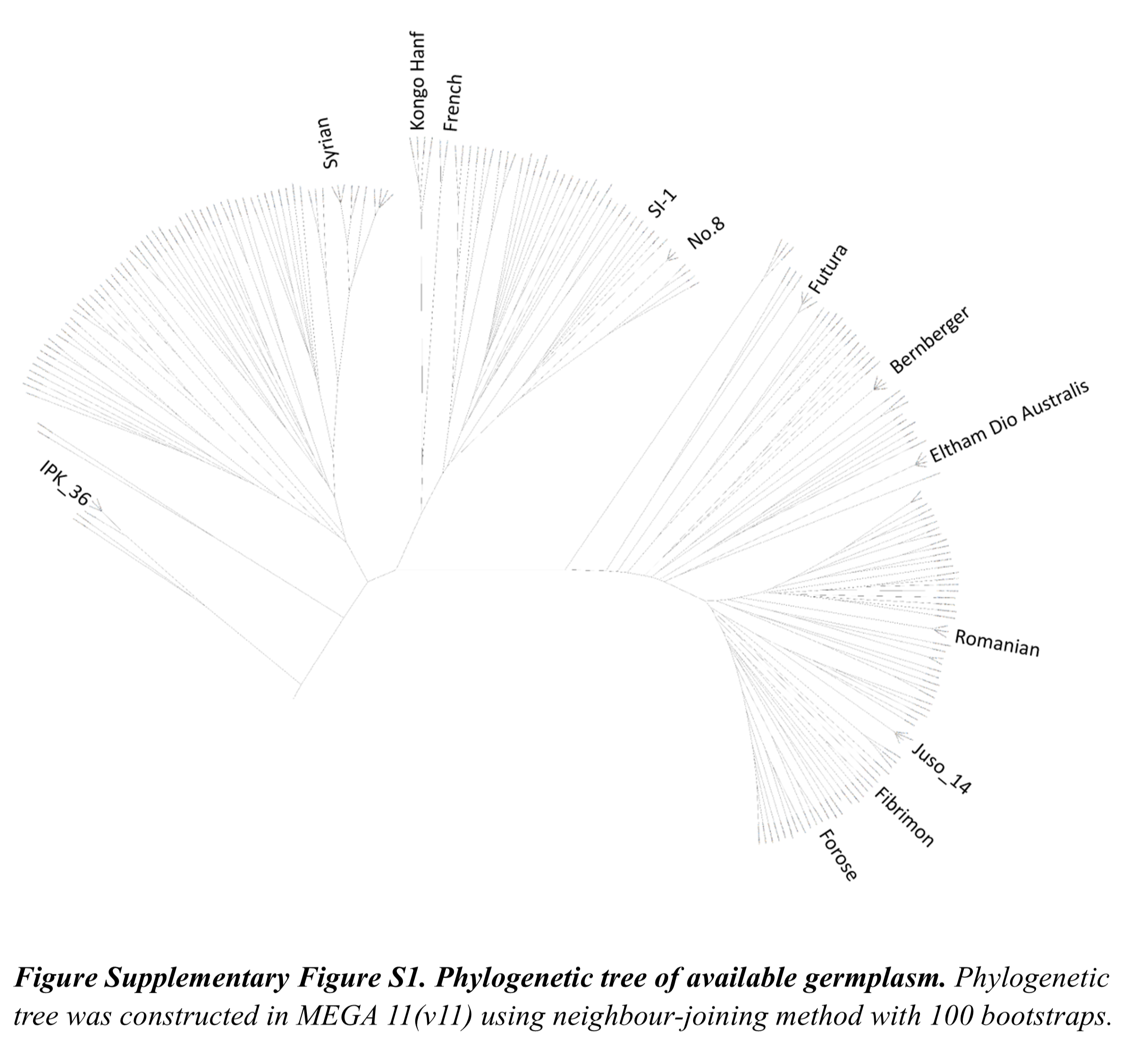

Supplement: Web_Material_uhag038 [file web_material_uhag038.zip › Supplementary_Figure_S1.PNG]

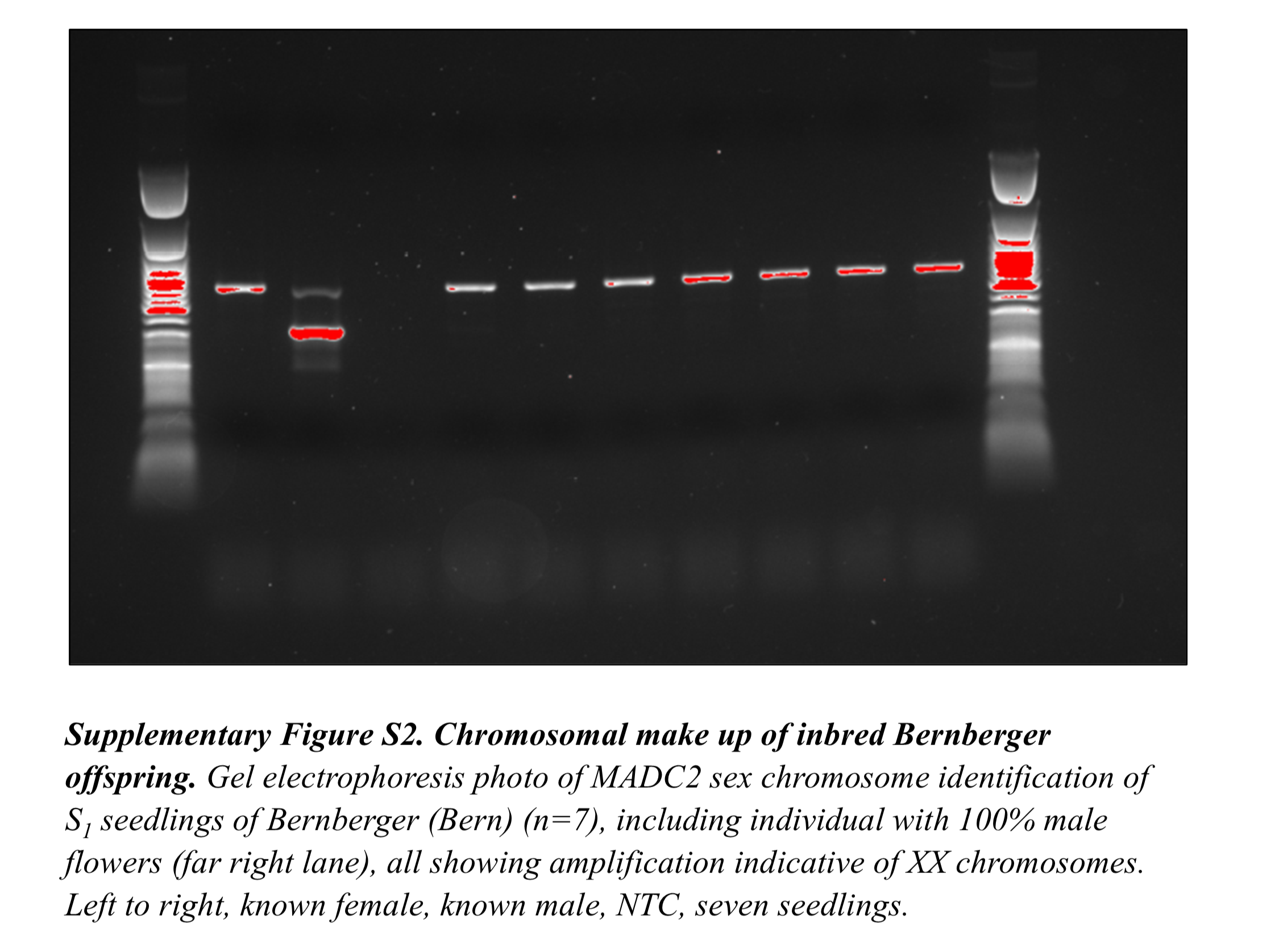

Supplement: Web_Material_uhag038 [file web_material_uhag038.zip › Supplementary_Figure_S2.PNG]

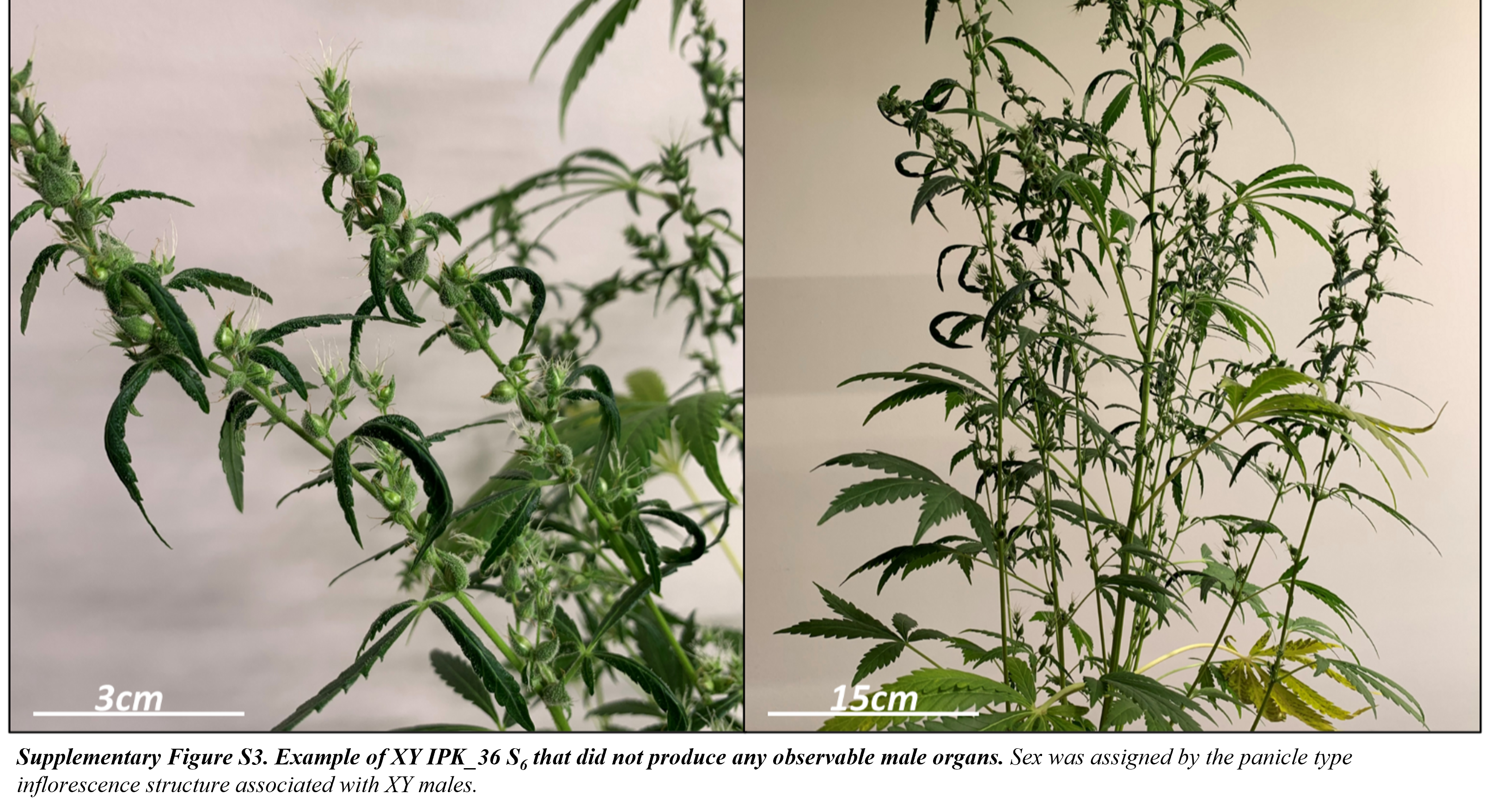

Supplement: Web_Material_uhag038 [file web_material_uhag038.zip › Supplementary_Figure_S3.png]
